# Lactate: The Mediator of Metabolism and Immunosuppression

**DOI:** 10.3389/fendo.2022.901495

**Published:** 2022-06-09

**Authors:** Yuanyuan Zhang, Zhao Zhai, Jiali Duan, Xiangcai Wang, Jinghua Zhong, Longqiu Wu, An Li, Miao Cao, Yanyang Wu, Huaqiu Shi, Jianing Zhong, Zhenli Guo

**Affiliations:** ^1^ The First Affiliated Hospital of Gannan Medical University, Ganzhou, China; ^2^ Key Laboratory of Prevention and Treatment of Cardiovascular and Cerebrovascular Diseases of Ministry of Education, Gannan Medical University, Ganzhou, China

**Keywords:** the Warburg effect, lactate, histone lysine lactylation, metabolic reprogramming, immunosuppression

## Abstract

The Warburg effect, one of the hallmarks of tumors, produces large amounts of lactate and generates an acidic tumor microenvironment *via* using glucose for glycolysis. As a metabolite, lactate not only serves as a substrate to provide energy for supporting cell growth and development but also acts as an important signal molecule to affect the biochemical functions of intracellular proteins and regulate the biological functions of different kinds of cells. Notably, histone lysine lactylation (Kla) is identified as a novel post-modification and carcinogenic signal, which provides the promising and potential therapeutic targets for tumors. Therefore, the metabolism and functional mechanism of lactate are becoming one of the hot fields in tumor research. Here, we review the production of lactate and its regulation on immunosuppressive cells, as well as the important role of Kla in hepatocellular carcinoma. Lactate and Kla supplement the knowledge gap in oncology and pave the way for exploring the mechanism of oncogenesis and therapeutic targets. Research is still needed in this field.

## Introduction

The Warburg effect, proposed by Otto Warburg in the 1920s, suggests that cancer cells predominantly relied on increased glycolysis in the presence of oxygen, accompanied by the generation of lactate and the acidic microenvironment (1). This process is also known as aerobic glycolysis. It has been thought that it is only the cancer cells that consume lots of glucose, but accumulating evidence has found that immune cells, predominantly myeloid cells, also do (2, 3). What is more interesting is that these cells continue to proliferate and establish immunosuppressive networks in the presence of lactate to gain unlimited immune escape potential (4). Thus, lactate acts as a mediator between metabolic reprogramming and immunosuppression (5). It is worth noting that in 2019, Zhang et al. proposed histone lysine lactylation (Kla), a mechanism with global influence, which provides a new research direction for exploring carcinogenesis and effective therapeutic targets (6). The liver is an organ that constantly metabolizes glucose, fat, and amino acids according to the needs of the body. The changes of related enzymes and metabolites are valuable to be used to evaluate liver function and predict the development of cancer in clinic (7–9).

## Metabolic Reprogramming to Produce Lactate

Glucose and glutamine are important sources of energy for tumor cells and immune cells, with glucose predominating. Nutritional competition occurs between two types of cells. Tumor cells consume large amounts of both substances, resulting in immune cell starvation and decreased antitumor immunity (10).

### The Warburg Effect

**Figure 1:**
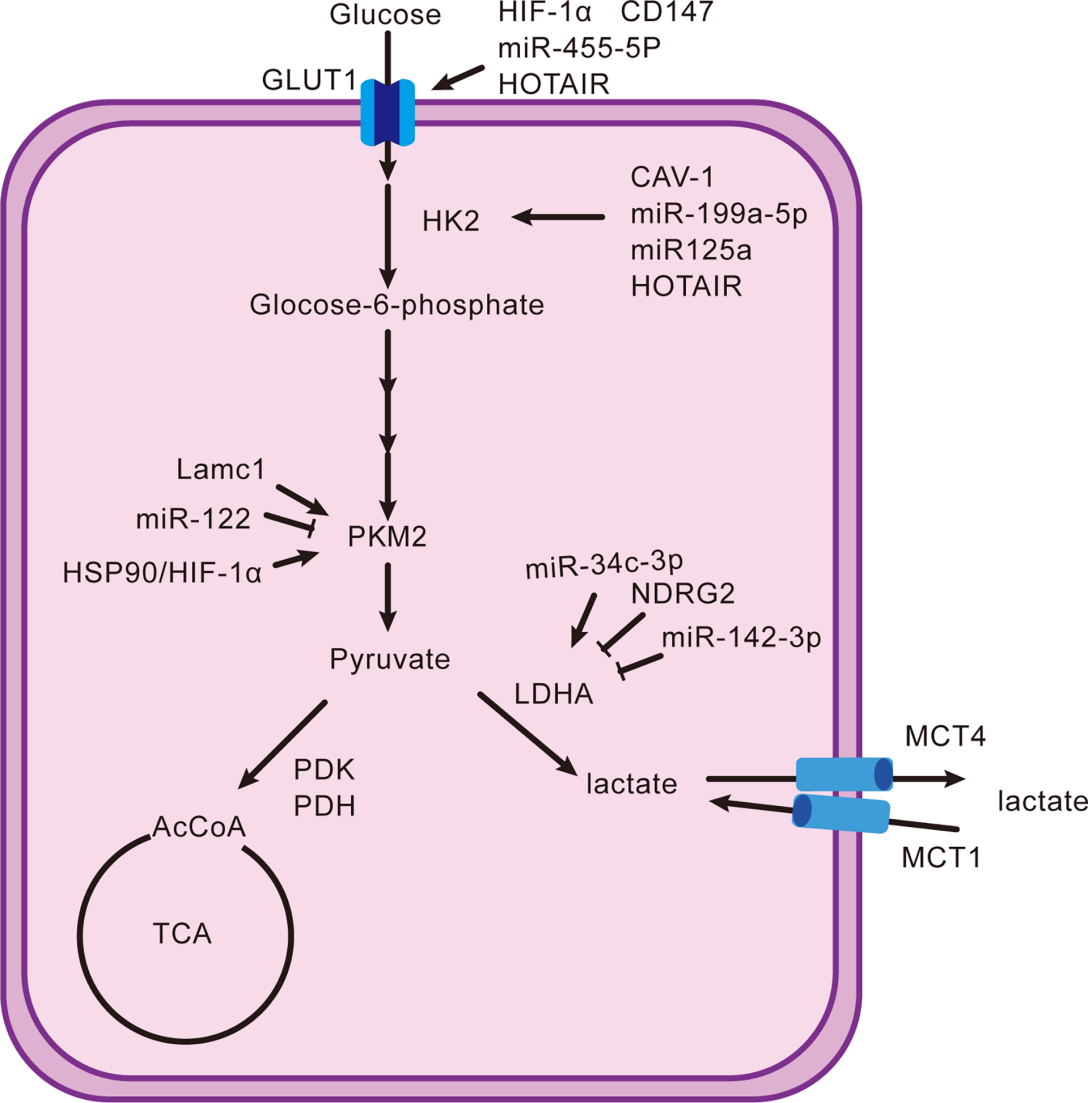
Production and reduction mechanism of lactate. It shows the classical intracellular pathway(s) that generates lactate and their upregulators or downregulators from the proteomic, genomic, and transcriptomic domains.

The end point of the Warburg effect is lactate regulated by many key enzymes. First, the glucose transporter (GLUT), which is expressed in most cells, is involved in transporting glucose into cells in metabolically active tissue. A variety of tumors highly express GLUT1 and have many regulatory factors (11). Hypoxia-inducible factor-1α (HIF-1α) and CD147 have positive relations with GLUT1, respectively (12, 13). MiR-455-5p also promotes the upregulation of GLUT1 by the IGF-1R/Akt/GLUT1 pathway (14). Long non-coding RNAs (lncRNAs), which were previously considered as “transcriptional noise” because of the non-protein-coding function, play a significant role in the development of tumors (15). For example, HOX transcriptional antisense RNA (HOTAIR) increases the expression of GLUT1 by activating the mTOR signaling pathway (16). Upregulating the GLUT1 and expediting glucose absorption are only the first step in lactate production. Second, hexokinase (HK) handles the conversion of glucose to glucose-6-phosphate. There are four types of HK, HK1–4, of which HK2 trumps others in hepatocellular carcinoma. Caveolin1 (CAV-1), miR-199a-5p, and miR-125a take effect (17–20). HOTAIR is also one factor by adsorbing miR-130a-3p without oxygen (21). Glucokinase, an isoenzyme of HK, is required for migration of regulatory T cells (Treg) (22). There are few studies on HK2, and focusing on it may lead to special findings. Moreover, pyruvate kinase 2 (PKM2) is the final rate-limiting enzyme regulating pyruvate accumulation. The Lamc1/PTEN/AKT pathway causes an increase in PKM2 expression, as does the downregulation of miR-122 (23, 24). The interaction between PKM2, heat shock protein 90 (HSP90), and HIF-1α is to stabilize PKM2 and induce aerobic glycolysis to inhibit cell apoptosis (25). In addition, lactate dehydrogenase (LDH), pyruvate dehydrogenase kinase (PDK), and pyruvate dehydrogenase (PDH) are the key enzymes that determine the destination of pyruvate. LDHA and LDHB encode LDH and form five types in different proportions: LDH1 (B4), LDH2 (AB3), LDH3 (A2B2), LDH4 (A3B), and LDH5 (A4) (26). Interestingly, LDH5 is more significantly expressed than other types in hepatocellular carcinoma. Thus, inhibition of LDHB transcription or induction of LDHA transcription is an essential mechanism that promotes the generation of lactate (27). Decreased miR-142-3p and N-myc downstream-regulated gene 2 (NDRG2) and elevated miR-34c-3p stimulate the upregulation of LDHA (28–31). The generated lactate continues to mediate the activity of PDK to phosphorylate PDH, causing a blocked entry of pyruvate into the tricarboxylic acid cycle (TAC) (27). On the one hand, it reduces the consumption of glucose by oxidative phosphorylation (OXPHOS), and on the other hand, it can cause the accumulation of pyruvate and indirectly facilitate the production of lactate. Differently, it is FoxP3 in Treg cells that impairs glycolysis and promotes OXPHOS that confer migratory and inhibitory functions on Treg cells (32). Finally, the acidic environment also depends on the pH-dependent monocarboxylate transport system (MCT), a tool for lactate to shuttle between cells and the microenvironment. The flow of lactate into and out of cells relies on MCT1 and MCT4, respectively (33). The transfer of lactate disrupts extracellular pH homeostasis, which not only affects the activity of enzymes but also participates in regulating immune cells. Lactate can also act as a substrate to energize cells, which is referred to as the “reverse Warburg effect” (34). In this way, the role of lactate is too diverse and complex to be ignored. **Figure 1** shows the production and regulation mechanism of lactate in hepatocellular carcinoma cells.

### Glutamine Metabolism

Glutamine metabolism has two main ways *in vivo*. On the one hand, it provides C–N (amide group) for the synthesis of protein, lipid, and nucleotide (35); on the other hand, metabolites such as aspartate and oxaloacetate can be converted to substances for glycolysis (36). Cancer cells contain high levels of glutaminase 1 (GLS1), which decomposes glutamine into glutamate. Targeting GLS1 can inhibit not only cell growth but also stemness characteristics (37–39). In addition, there was a possible inhibitory relationship between glutamine metabolism and glycolysis. However, glucose is not severely limited because of the presence of resident cells (such as immune cells and endothelial cells), which can enhance glucose intake (2). More unexpectedly, glutamine metabolism also promotes cell growth and suppresses immunity because of lactate (40). Therefore, targeting glutamine is a meaningful therapeutic strategy that deserves to explore more mechanisms.

## Lactate Facilitates the Assembly of Immune Escape Network

**Figure 2:**
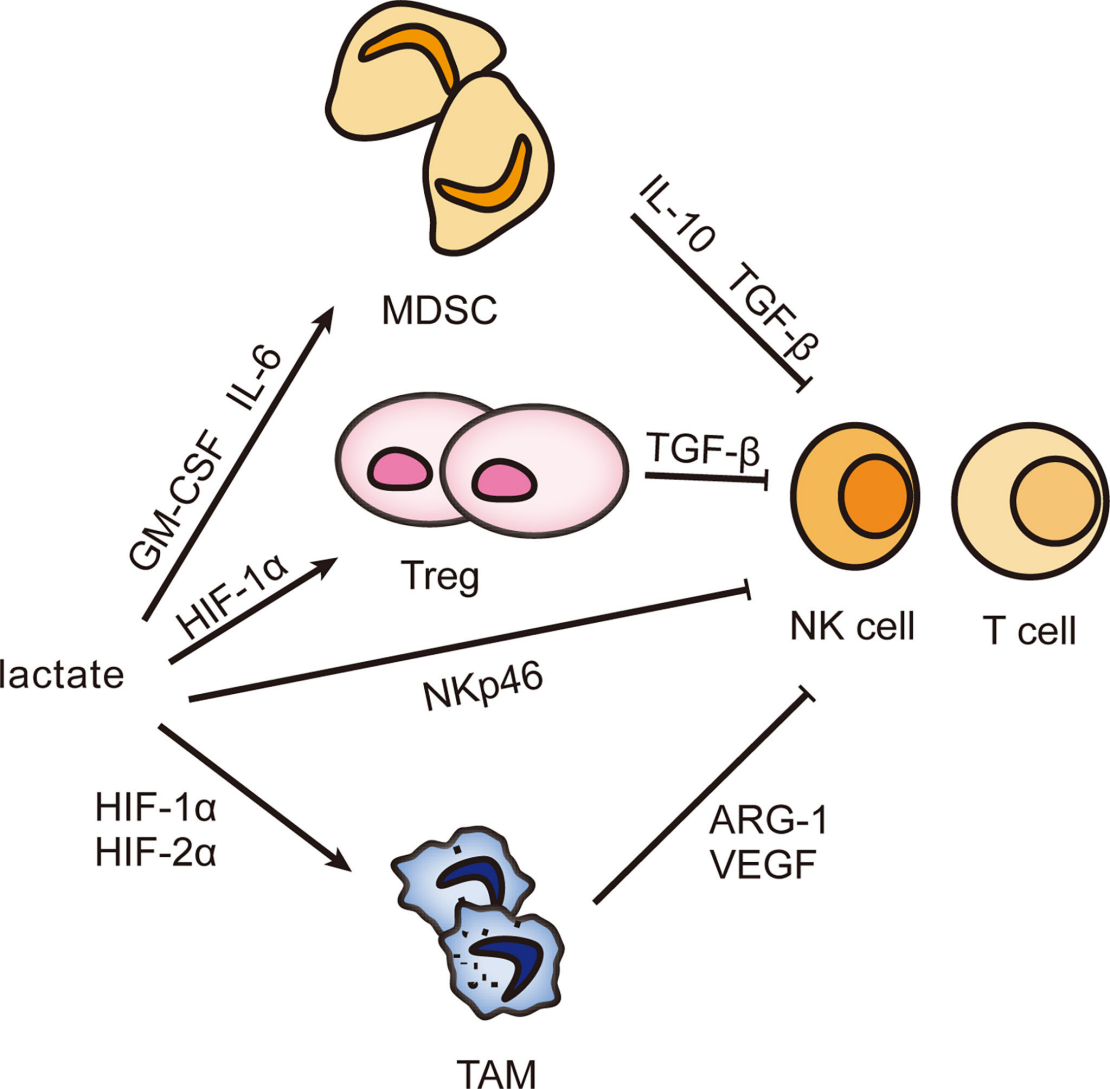
Effect of lactate on infiltrating immune cells. The accumulation of lactate can induce the differentiation of MDSC, Treg, and TAM and stimulate their biological activities and then secrete immunosuppressive factors to inhibit the immune response of NK cells and T cells, helping tumor cells escape immune surveillance and gain unlimited growth potential.

The liver possesses unique immune properties containing large populations of NK cells, cytotoxic CD8^+^ T cells, and others, but the incidence of hepatocellular carcinoma remains high, most possibly because an external environment suppresses immunity (41). Lactate can facilitate the assembly of an immune escape network to make the proliferation of tumor cells uncontrolled (42, 43). Lactate-mediated immune escape networks are shown in **Figure 2**.

### Myeloid-Derived Suppressor Cells

Myeloid-derived suppressor cells (MDSCs) comprise a group of heterogeneous cells derived from immature bone marrow. MDSCs are the precursor of mature monocytes, dendritic cells, and granulocytes. However, they are an important assistant to favor tumor proliferation and inhibit immune response (44, 45). Multiple cytokines prevent MDSCs from differentiating and maturing, then MDSCs expand and migrate. Lactate is one (**Table 1** shows the mechanism of other factors) (55–57). M6A demethylase ALKBH5 ensures the m6A methylation level and RNA stability of MCT4, followed by lactate transporting out of cells with the help of MCT4 and stimulating MDSC increase (58). The knockdown of LDHA reconfirms the role of lactate with reduced MDSCs and weakened cytolytic function of NK cells (59). How does lactate assume this responsibility? Lactate induces the accumulation of MDSCs through granulocyte-macrophage colony-stimulating factor (GM-CSF) and IL-6. Besides, lactate inhibits the expression of activated receptor NKp46 in NK cells (59). This shows that lactate can directly and indirectly inhibit the antitumor activity, and thus the more vigorous the tumor metabolism, the poorer the immune function. More interestingly, MDSCs are necessary for tumor-associated macrophage (TAM) accumulation. When MDSCs migrate to the tumor site, its CD45 phosphatase activity increases because of hypoxia and local acidification, which inhibits STAT3 and thus promotes the conversion of MDSCs to TAMs (60). In addition, lactate-induced HIF1α can also promote the differentiation of MDSCs into TAMs by regulating the expression of inducible nitric oxide synthase (iNOS) and arginase-1 (ARG1), which is helpful to inhibit adaptive immunity (61–63). In summary, lactate is involved in multiple pathways to suppress the antitumor immunity, so could lactate be a target for tumor treatment? During radiotherapy for pancreatic cancer, lactate plays an important role in enhancing the immunosuppressive phenotype of MDSC through the G-protein-coupled receptor 81 (GPR81)/mTOR/HIF-1α/STAT3 pathway. Therefore, targeting lactate may provide a unique prospect for improving the radiosensitivity of pancreatic cancer (64).

**Table 1:** Other factors stimulate MDSC accumulation in hepatocellular carcinoma.

Molecule	Primary mechanism of action	Reference
HSC	Activation of the COX2/PGE2/EP4 and SDF-1/CXCR4 signaling pathways	(46, 47)
TAF	Activation of the SDF-1α/CXCR4 and IL-6/STAT3 pathways	(48)
AMPK	Suppression of the JAK/STAT, NF-kB, C-EBPβ, and CHOP signaling pathways	(49)
HIF-1α	Conversion of extracellular ATP to 5′-AMP through its direct transcriptional target, ENTPD2, and promotion of the interaction between CCL26 and CX3CR1	(50, 51)
PIWIL1	Induction of P38MAPK signaling	(52)
PKM2	Activation of PI3K-AKT and JNK signaling pathways and upregulation of HIF-1α expression	(53)
RIP3	Promotion of the interaction between CXCL1 and the cognate receptor CXCR2	(54)

### Tumor-Associated Macrophage

In the 1980s, the fact that monocytes differentiated into macrophages under the recruitment of chemokines was confirmed (65). Solid tumors exhibit hypoxic areas within the tumor mass. Macrophages are attracted to these hypoxic tumor sites by various chemodynamic stimuli secreted by tumor cells under hypoxic pressure, and their movement is directly impaired, causing tumor-associated macrophage (TAM) to become trapped at the site of the ischemic tumor (66, 67). “Activated macrophages” induce vascular proliferation, and “non-activated macrophages” do not (68). Now these two states have been named selectively activated macrophages (M2) and classically activated macrophages (M1), respectively. TAMs are broadly heterogeneous, and their functional status depends on the conditions of exposure (69). For example, Toll-like receptor agonists, interferon-γ (IFN-γ), and lipopolysaccharide (LPS) promote the phenotype of M1, while IL-4, IL-13, IL-10, transforming growth factor-β (TGF-β), and lactate are inducers of M2 (70–72).

Lactate promotes M2 by stabilizing the expression of HIF-1α, and in this process ARG1 and vascular endothelial growth factor (VEGF) are increased, which are closely related to wound healing, angiogenesis, and tissue remodeling (73). In addition, HIF-2α is also implicated. Lactate negatively regulates transcription factor EB (TFEB) by activating mTORC1 to reduce the expression of ATP6V0d2, which mediates the degradation of HIF-2α by lysosomes, ensuring the activated state of M2 (74). Epigenetics also contributes to an important mechanism. Histone lysine lactylation (Kla) has been shown to promote M2 activation, but the exact mechanism is not well understood (6). M2 secretes IL-10 and TGF-β in response to lactate stimulation to weaken the anticancer ability of NK cells and lymphocytes (73). Thus, it is established that lactate affects immune function through M2 and lactate could be a therapeutic target. Machilin A inhibits cell growth and M2 polarization by decreasing lactate production (75). When macrophages were cultivated in acidic pH conditions, HIF-1α, ARG1, and VEGF were found increased. So is it lactate or acidic conditions that are required to promote tumor development? This deserves further study and exploration.

### Regulatory T Cells

Lactate also induces aggregation of regulatory T cell (Treg), the phenotype of CD4+CD25+FoxP3+ T cell. Its metabolic pathway varies with activity and function (13, 76). First, the high-speed glycolysis of Treg driven by the mTOR signal will not only metabolically restrict antitumor cells such as T lymphocytes, which is called T-cell starvation, but provide energy for Treg proliferation and migration from the thymus to the tumor periphery (10, 22). In addition, lactate-induced HIF-1α activates the CCL20/CCR6 axis by inducing myeloid trigger receptor-1 (TREM-1) expression in TAM, attracting aggregation and initiating immunosuppressive effects of Treg (77). Most unexpectedly, Treg’s activity requires a switch from glycolysis to OXPHOS, when forehead box P3 (FoxP3) comes into play. It not only inhibits glycolysis by suppressing the oncogene Myc but also regulates LDH to promote the conversion of lactate to pyruvate and even drives lipid oxidation metabolism to promote OXPHOS, which endows Treg with inhibitory activity (32, 78, 79). A stable expression of FoxP3 is associated with epigenetic modifications, particularly methylation. Promoters and CpG islands are the main methylation sites of FoxP3. The methylation level is negatively correlated with the proportion of Treg, which means that the lower the methylation level of FoxP3, the higher the proportion of Treg cells and the higher the tumor malignancy (80). TGF-β can induce FoxP3 demethylation (81). Treg performs glycolysis, OXPHOS, and fat metabolism. Different activities and functions utilize different metabolisms, which adds some complexity to Treg.

## Histone Lysine Lactylation

In 2019, a new epigenetic modification of gene transcription, histone lysine lactylation (Kla), was proposed, which is based on lactate and lysine (6). This finding highlights how lactate has an advanced effect on tumor progression, and provides a mechanism by which some pathways of angiogenesis, migration, and metastasis may be activated. Kla fills an important gap in our understanding of various physiopathologies (such as cancer) that are closely related to lactate. Using M1 macrophage polarization as a model, it was confirmed that Kla regulated gene expression. Because of a low level of ARG1, M1 macrophages metabolize arginine through nitric oxide synthase (NOS) to produce nitric oxide (NO) to kill pathogens, while M2 macrophages have a higher level of ARG1, which produces ornithine to promote wound healing (82). After inhibiting LDH, the yield of lactate and the degree of Kla decreased, including the Kla markers on ARG1 promoters. Then M1 macrophages were treated with exogenous lactate to find that the global Kla and ARG1 expression increased (6). These conclusions have also been confirmed in melanoma and lung cancer (6). It scientifically and rigorously verified the positive role of lactate and Kla in inducing the polarization of M1 macrophage to M2 macrophage.

Kla can cause carcinogenic signals and represent a clear target for tumor therapy. Convincing evidence suggests that Kla promotes the transcription of YTHN6-methyladenosine RNA-binding protein 2 (YTHDF2). YTHDF2 recognizes the M^6^ A modification sites on PER1 and TP53 and promotes their degradation, thus promoting the occurrence of melanoma (83). This study revealed the cancer-promoting mechanism of Kla, confirmed the interaction between Kla and M^6^ A methylation for the first time, and provided a new therapeutic target to treat melanoma. In addition, Kla has also been found to regulate gene expression in non-small cell lung cancer (NSCLC). After treatment of NSCLC cells with lactate, the degree of Kla and HIF-1α increases and the transcription of HK-1, glucose 6-phosphate dehydrogenase (G6PD), and PKM decreases, which jointly regulates the metabolism of cancer cells (84). This further confirms the important role of Kla in cancer.

## Conclusion

To sum up, lactate is the product of aerobic glycolysis. It is used as an energy source for many kinds of cells, including tumor cells, and as a signal molecule to damage the immune response directly or indirectly. Kla, a kind of posttranslational modification, can regulate gene expression. Reducing the concentration of lactate and Kla modification contributes to restoring the physiological function of the body, so what promising strategies can achieve this effect? One is to block the production pathway of lactate by inhibiting the key enzymes of glycolysis and glutaminolysis, and the other is to block the transport of lactate (85–87). Ideally, lactate concentration and anticancer immunity return to the physiological state (88). However, in view of the diversity of enzymes, it is critical to find an enzyme that can regulate lactate to the maximum extent. In addition, how to solve the damage to normal cells is also a big problem. Therefore, targeting lactate and Kla is a frontier treatment, but there is a long way to go.

## Author Contributions

YZ: original draft preparation. ZZ, JD, MC, and YW: software,figure, and table. XW, JZ, LW, and AL: revising the manuscript critically. HS, JZ, and ZG: reviewing and editing. All authors contributed to the article and approved the submitted version.

## Funding

This project was supported by the Major Program of the National Natural Science Foundation of China (No. 82060363) and Science and Technology Plan of Jiangxi Provincial Health and Health Commission (SKJP220203570).

## Conflict of Interest

The authors declare that the research was conducted in the absence of any commercial or financial relationships that could be construed as a potential conflict of interest.

## Publisher’s Note

All claims expressed in this article are solely those of the authors and do not necessarily represent those of their affiliated organizations, or those of the publisher, the editors and the reviewers. Any product that may be evaluated in this article, or claim that may be made by its manufacturer, is not guaranteed or endorsed by the publisher.

## Abbreviations

Kla, histone lysine lactylation; GLUT, glucose transporter; HIF-1α, hypoxia-inducible factor-1; lncRNAs, long non-coding RNAs; HOTAIR, HOX transcriptional antisense RNA; HK, hexokinase; CAV-1, Caveolin1; Treg, regulatory T cells; PKM2, pyruvate kinase 2; HSP90, heat shock protein 90; LDH, lactate dehydrogenase; PDK, pyruvate dehydrogenase kinase; PDH, pyruvate dehydrogenase; NDRG2, N-myc downstream regulated gene 2; TAC, tricarboxylic acid cycle; OXPHOS, oxidative phosphorylation; MCT, pH-dependent monocarboxylate transport system; GLS1, glutaminase1; MDSC, myeloid-derived suppressor cell; GM-CSF, granulocyte-macrophage colony-stimulating factor; iNOS, inducible nitric oxide synthase; ARG1, arginase-1; GPR81, G-protein-coupled receptor 81; TAM, tumor-associated macrophage; M2, selectively activated macrophages; M1, classically activated macrophages; IFN-γ, interferon-γ; LPS, lipopolysaccharide; TGF-β, transforming growth factor-β; VEGF, vascular endothelial growth factor; TFEB, transcription factor EB; TREM-1, myeloid trigger receptor-1; FoxP3, forehead box P3; NOS, nitric oxide synthase; NO, nitric oxide; YTHDF2, YTHN6-methyladenosine RNA-binding protein 2; NSCLC, non-small cell lung cancer; G6PD, glucose 6-phosphate dehydrogenase

## Glossary

Kla: histone lysine lactylation
GLUT: glucose transporter
HIF-1a: hypoxia-inducible factor-1
lncRNAs: long non-coding RNAs
HOTAIR: HOX transcriptional antisense RNA
HK: hexokinase
CAV-1: Caveolin1
Treg: regulatory T cells
PKM2: pyruvate kinase 2
HSP90: heat shock protein 90
LDH: lactate dehydrogenase
PDK: pyruvate dehydrogenase kinase
PDH: pyruvate dehydrogenase
NDRG2: N-myc downstream regulated gene 2
TAC: tricarboxylic acid cycle
OXPHOS: oxidative phosphorylation
MCT: pH-dependent monocarboxylate transport system
GLS1: glutaminase1
MDSC: myeloid-derived suppressor cell
GM-CSF: granulocyte-macrophage colony-stimulating factor
iNOS: inducible nitric oxide synthase
ARG1: arginase-1
GPR81: G-protein-coupled receptor 81
TAM: tumor-associated macrophage
M2: selectively activated macrophages
M1: classically activated macrophages
IFN-g: interferon-g
LPS: lipopolysaccharide
TGF-b: transforming growth factor-b
VEGF: vascular endothelial growth factor
TFEB: transcription factor EB
TREM-1: myeloid trigger receptor-1
FoxP3: forehead box P3
NOS: nitric oxide synthase
NO: nitric oxide
YTHDF2: YTHN6-methyladenosine RNA-binding protein 2
NSCLC: non-small cell lung cancer
G6PD: glucose 6-phosphate dehydrogenase
